# Increasingly cautious sampling, not the black colouration of unpalatable prey, is used by fish in avoidance learning

**DOI:** 10.1007/s10071-023-01815-9

**Published:** 2023-07-28

**Authors:** Mikołaj Kaczmarski, Jan M. Kaczmarek, Krzysztof Kowalski, Karol Borowski, Jacek Kęsy, Janusz Kloskowski

**Affiliations:** 1 Department of Zoology, Poznań University of Life Sciences, Wojska Polskiego 71 C, 60-625 Poznań, Poland; 2 Landscape Parks of Greater Poland Voivodeship, Piekary 17, 61-823 Poznań, Poland; 3 Department of Vertebrate Zoology and Ecology, Institute of Biology, Faculty of Biological and Veterinary Sciences, Nicolaus Copernicus University in Toruń, Lwowska 1, 87-100 Toruń, Poland; 4 Chair of Plant Physiology and Biotechnology, Institute of Biology, Faculty of Biological and Veterinary Sciences, Nicolaus Copernicus University in Toruń, Lwowska 1, 87-100 Toruń, Poland

**Keywords:** Aposematism, Common toad, Predation, Tadpoles, Warning colouration

## Abstract

The efficiency of aposematic colouration of prey is based on the innate bias or facilitation of avoidance learning of predators. In many toxic bufonids, larvae are uniformly black, which is considered a warning signal. We compared fish predation on normal (black) and ‘transient albino’ (greyish) common toad *Bufo bufo* tadpoles that did not differ in toxicity or activity. In a two-stage experiment, each fish was presented with tadpoles of one colour in the first trial and the other colour in a subsequent trial. While tadpoles sampled by fish were typically not ingested, some died from injuries. The attack rate did not differ between tadpole phenotypes nor trials, irrespective of which phenotype was the first exposed to the fish. However, during the second trial, the sampled tadpoles, independent of colouration, were mouthed by fish for shorter periods and tadpole mortality decreased. The duration of mouthing also declined with an increasing number of attacks during subsequent trials. We conclude that in single-species prey populations, black tadpole colouration is not a warning signal as it does not accelerate predator learning about prey unprofitability. Our results indicate that with growing experience, predators sample potentially toxic prey more cautiously. This may explain why natural selection does not eliminate aposematic morphs even if predators continuously sample conspicuous prey.

**Supplementary Information:**

The online version contains supplementary material available at 10.1007/s10071-023-01815-9.

## Introduction

Aposematic traits, like distinctive colouration, pattern, odour or sound, are warning signals to reduce predation risk. Such signals must be conspicuous before an attack begins and are considered a primary defence mechanism of the prey, advertising its unprofitability to predators. The features evoking aversion in the predator are considered secondary defences (Ruxton et al. 2004). A predator may either show innate bias against warning traits (Sillén-Tullberg 1985; Marples and Roper 1996; Rowe and Guilford 1996; Lindström et al. 1999) or learns to avoid a given type of prey due to the combination of both sets of traits (Sillén-Tullberg 1985; Speed 2000; Gamberale-Stille and Guilford 2004). In a learning process, the chances of the unpalatable prey being injured or killed should decrease (Ruxton et al. 2004; Rojas et al. 2017). However, predator naivety and/or deceptive (auto)mimicry result in predator uncertainty towards prey, driving the need to verify palatability. Predator learning via prey sampling (Skelhorn and Rowe 2006) poses an evolutionary problem of the aposematic prey's costs of educating the predators, especially as conspicuous traits increase prey detection rate (Wiklund and Järvi 1982; Ruxton et al. 2004; Puurtinen and Kaitala 2006; Skelhorn and Rowe 2006).

Conspicuous colours, such as black, red or yellow and their combinations, may have a warning function (Schuler and Roper 1992; D’Heursel and Haddad 1999) because they provide a high contrast against natural backgrounds (Gamberale-Stille 2001; Stevens and Ruxton 2012). In many toad species (Bufonidae) that possess chemical defences and are avoided by vertebrate predators, especially fish (Daly et al. 1987; Letnic et al. 2008; Kowalski et al. 2018), tadpoles are uniformly black. This black colouration is considered to be aposematic (Peterson and Blaustein 1991; Wells 2013), although alternative explanations have also been discussed (Guilford 1988). The effectiveness of black as an aposematic colouration of tadpoles may vary depending on the predator identity (D’Heursel and Haddad 1999; Gontijo et al. 2018). The potential aposematic function of contrasting colours is poorly understood in aquatic organisms as the refractive index of water is significantly different to air. Vision below the water surface depends on its properties, including transparency and light intensity (Vogel and Beauchamp 1999; Mazur and Beauchamp 2003). Black is assumed to be conspicuous under wide light conditions underwater (Levine et al. 1980; but see: Kinney et al. 1967), so it has the potential to act as an aposematic signal in aquatic animals. Fishes, the dominant aquatic predators to whom bufonid tadpoles appear invulnerable, have a well-developed sense of sight and see more colours than humans (Neumeyer 1992; Neumeyer and Mora-Ferrer 2001).

Our aim was to understand how predatory fish avoid unpalatable prey, in particular, whether black colouration of bufonid tadpoles is an aposematic signal. Experimental testing of the role of potential warning colouration in living aquatic animals is challenging because of difficulties with non-invasively manipulating animal colouration in aquatic environments. We circumvented this issue in predation trials using ‘transient albino’ (non-uniformly greyish coloured) and normal black tadpoles of the common toad *Bufo bufo* Linnaeus, 1758 that did not differ in their level of chemical defences. We predicted that if black colouration was a genuine warning signal, the fish would more rapidly learn to avoid black-coloured prey than the greyish, but similarly unpalatable, conspecifics. We compared the frequency of fish attacks on the two phenotypes in two consecutive trials (tadpoles of each phenotype were separately presented to fish in one of the trials), prey handling time and tadpole survival during each trial.

## Materials and methods

### The experiment

Large portions of two freshly laid egg strings of the common toad, one normally (black) coloured and one albino (white), were collected from a suburban pond near Poznań (52° 20′13.8″ N 16° 58′47.2″ E) and separately stored in aged tap water. After hatching, 100 tadpoles were randomly selected for each phenotype. The two groups were reared independently under uniform conditions in 100-L containers up to Gosner stage 25 (Gosner 1960); the same group sizes were used to standardise toxin production by tadpoles in response to conspecific density (Bókony et al. 2018).

We used the wild phenotype of goldfish, *Carassius auratus* Linnaeus, 1758, as a predator. *Carassius auratus* is a cyprinid native to Eastern Asia but widely introduced elsewhere (Savini et al. 2010). The goldfish is a generalist forager with a varied diet, including plankton, bottom-dwelling invertebrates and amphibian larvae (Monello and Wright 2001). Goldfish presence can affect amphibians in complex ways, invoking strong non-consumptive effects (Winandy and Denoël 2013, 2015). Owing to its omnivorous diet and ecosystem engineering abilities, *C. auratus* is an ecologically relevant model species representative of a large group of widely spread carp fishes, such as *Carassius* spp. and *Cyprinus* spp. (Richardson et al. 1995; Kloskowski 2009; Huang et al. 2020). Colour vision in this species is tetrachromatic (Neumeyer 1992). The goldfish (age 1 + fish) were obtained from a fish retailer. The fish were reared in semi-natural ponds without experience with common toad tadpoles (the only amphibian with black and toxic larvae in the region) until their first winter, after which they were kept in artificial conditions. The mean total length of the fish was 89.3 ± 1.5 mm (mean ± SE). At this size cyprinid fishes attain the ability to prey on freely-moving tadpoles in mid and late developmental stages (cf. Kloskowski 2009); hence 1 + spring is the period goldfish learn about the palatability of tadpoles in natural conditions.

For one week before the experiment, the fish were stored outdoors in a 120 × 100 cm^2^ tank and fed granulated feed and Chironomidae bloodworm larvae ad libitum. Twenty-four hours before the experiment, the goldfish were placed individually in plastic 39 × 28 × 14 cm plastic containers filled with approximately 10 L of aged tap water (temperature 18 °C), the bottom covered with commercial aquarium sand. To standardize hunger levels, fish were not fed during this period, except for receiving three bloodworm larvae of a similar size one hour before the trials. Five tadpoles of the same phenotype (either black or albino) were introduced into each container. As the transient albino tadpoles darken progressively over time (Henle et al. 2017), they were greyish at Gosner stage 25, thus, resembling non-aposematic tadpoles (Peterson and Blaustein 1991; Wells 2013). Throughout development, the differences in body colouration between the phenotypes were visible to a human observer (see Suppl. I). Using earlier stage tadpoles would provide larger differences in body colouration between groups; however, white colouration could also potentially function as an aposematic signal. Experimental treatments consisted of two subsequent 3-h trials, 10 min apart. Each tested fish was presented with both prey phenotypes (either first with albino and then black tadpoles or the reverse sequence, 9 fish individuals per each sequence of tadpole phenotype presentation, double trials, altogether 36 trials). Fish and tadpole behaviour was recorded using a Sony HDR-AS50 camera. The number of fish attacks (tadpole captures) during each trial was counted. The duration of prey handling (“mouthing”) was assessed using a stopwatch.

Since prey activity can alter predation rates (Gunzburger and Travis 2005), tadpole activity was assessed by recording the number of tadpoles swimming at the moment of observation. Activity counts were done near the beginning and in the middle of trials, each consisting of five repeated counts every minute from 15 to 19 min after trial onset, and 91 to 95 min, respectively. The mean proportion of active to non-active tadpoles at the beginning and in the middle of the trial was used in the analyses.

### Analysis of toxin content

In toad tadpoles, the antipredator defences are based on bufadienolides and proteins present in the skin (Lawler and Hero 1997; Crossland and Alford 1998; Crossland 2001; Üveges et al. 2017; Bókony et al. 2018; Kowalski et al. 2018). Liquid chromatography-electrospray ionisation tandem mass spectrometry was applied to identify the five most common and abundant bufadienolides (bufalin, bufotalin, cinobufagin, cinobufotalin and resibufogenin; for details, see Suppl. I). Mass-corrected bufadienolide quantity was calculated by dividing the concentration of each bufadienolide by the dry mass of individual tadpoles. For analyses, the values of all compounds were summed to estimate the total amount of bufadienolides per individual (Bókony et al. 2018).

### Statistical analysis

Linear mixed models were applied to assess the predatory behaviour of fish and tadpole survival. As the same fish individuals were used twice during the trials, fish identity was fitted as a random term. In all preliminary models, the colouration of tadpoles (black or albino), trial order (first or second trial) and sequence of tadpole phenotype presentation (which phenotype was presented first) were entered as fixed factors. Frequency of fish attacks on tadpoles (the total number of attacks during the trial) and duration of mouthing the prey were assessed using residual maximum likelihood models (REML). Residual plots were visually evaluated to ensure that each dataset met the assumption of normally distributed residual errors. The survival of tadpoles was analysed using generalised linear mixed models (GLMM) with a logit link and binomial distribution. The number of tadpole survivors was treated as a binomial response; the initial number of tadpoles constituted the binomial denominator. In the REML models, significance of the fixed terms was determined by the F statistics, and in the binomial models, by the Wald test. Model estimates were based on full models, except that the sequence of phenotype presentation and interaction terms (all P ≥ 0.275) were omitted due to non-significance. However, we also report minimal (backward simplified) models (cf. Forstmeier and Schielzeth 2011) when removing a highly insignificant predictor changed the significance value of another predictor to P < 0.05. All statistics were run in GenStat 15.1 (VSN International Ltd).

## Results

No significant differences were found between black and albino tadpoles in the total amount of the five analysed bufadienolides per body mass (t test, t_18_ = 1.65, P = 0.115, mean ± SE 654.0 ± 56.4 µg/g vs 507.6 ± 68.2 µg/g, respectively). Also, black and albino tadpoles did not differ in activity levels at the beginning (t_34_ = 1.35, P = 0.187) or the end of trials (t_34_ = 0.41, P = 0.683).

**Fig. 1:**
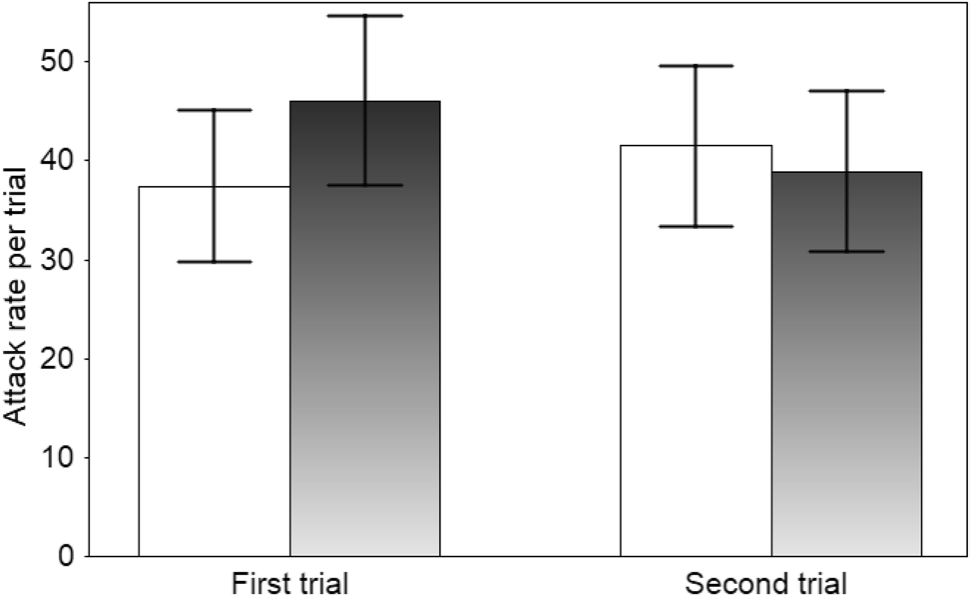
The mean (± SE) frequency of fish attacks per 3 h trial on normal black (filled bars) and transient albino (open bars) toad tadpoles

**Fig. 2:**
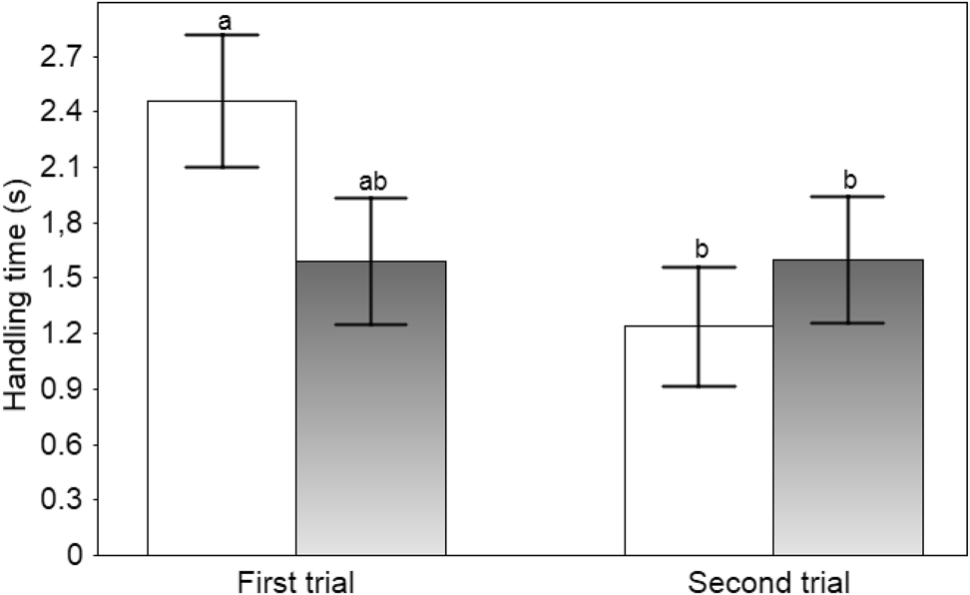
The mean (± SE) duration of prey mouthing by fish presented normal black (filled bars), and transient albino (open bars) toad tadpoles during 3 h trials. Different letters indicate significant difference at P < 0.05 (LSD test)

The fish attacked tadpoles in all of the trials (range 7–89 attacks per trial). The frequency of fish attacks did not differ between tadpole phenotypes nor for trial order (first or second) (Table 1, Fig. 1). The duration of prey handling (Fig. 2; post-hoc least-significant-difference (LSD) test showed that the effect was mainly explained by a strong decline in the duration of mouthing of the albino tadpoles) and the mortality of tadpoles, while not affected by tadpole phenotype, were lower in the second than the first trial (Table 1). As tadpole phenotype did not affect prey handling time by fish, an additional GLMM was run on the data from combined trials with the sequence of attacks (omitting a small number of immediately repeated attacks on the same prey) as a single fixed factor. The duration of prey mouthing declined with an increasing number of attacks during subsequent trials (F_1, 871.5_ = 7.45, P = 0.006). Most tadpole deaths were due to injuries from fish attacks (in total, ten during the first trial and three during the second), and only one tadpole was consumed.

**Table 1:** Results of the fixed effects portion of linear mixed models relating the frequency and duration of fish attacks (residual maximum likelihood models) and tadpole survival (binomial generalised linear mixed models) to tadpole phenotype (black vs transient albino) and trial order (first or the second trial)

Parameter	Fixed factor	F/Wald	d.f	P	Effect (SE/SED)
Attack frequency	Tadpole phenotype (black, albino)	0.32	1, 16.4	0.578	2.98 (5.26)
	Trial order (first, second)	0.06	1, 16.0	0.815	− 1.22 (5.15)
Attack duration	Tadpole phenotype (black, albino)	0.39	1, 1426.7	0.335	− 0.24 (0.25)
	Trial order (first, second)	5.57	1, 1424.4	0.021	− 0.57 (0.25)
Tadpole survival	Tadpole phenotype (black, albino)	0.01	1	0.938	− 0.05 (0.68)
	Trial order (first, second)	3.37	1	0.066	− 1.35 (0.73)
Tadpole survival (minimal model)	Trial order (first, second)	3.86	1	0.049	− 1.36 (0.69)

Fish identity was entered as a random term. Non-significant interaction terms were excluded. Effect estimates are reported with standard errors; for the binomial survival models, standard errors of differences are presented

## Discussion

Avoiding toxic prey can significantly increase individual fitness; hence, quick yet efficient learning about aposematism should be adaptive in predators (Glendinning 2007; Rowland et al. 2017). Aposematic signals may accelerate this learning process to the advantage of both predator and prey (D’Heursel and Haddad 1999; Mappes et al. 2005; Ruxton et al. 2008). In our study, the black colouration of tadpoles did not significantly reduce the attack rate and the prey handling time by fish predators relative to the greyish (albino) tadpoles. We infer that when prey populations consist of a single species, the black body colour does not function as a warning signal in the aquatic environment. We did not test the alternative aposematic function of the black colouration, i.e., whether it would be effective in discriminating toxic tadpoles from undefended mimics for a predator; however, the effects of visual signals and possible chemical species-recognition cues would need to be separated (Holen 2013) to determine which of these factors is more important in model-mimic discrimination. Here, given their similar toxin levels, we assumed that any chemical warning signals did not differ between the two phenotypes of this species.

Fish predators are known to avoid unpalatable bufonid tadpoles (Voris and Bacon 1966; Kruse and Stone 1984; Lawler and Hero 1997), but the processes managing their recognition of prey defences remain poorly understood. The rate of predator learning and the survival of unpalatable tadpoles may depend on the relative abundance of otherwise similar but palatable prey, as well as the predator hunger levels (Nelson et al. 2011; Kaczmarek et al. 2018; Kaczmarek et al. 2020; cf. Lindström et al. 2004; Rowland et al. 2010). We found that learning in fish was based on tasting prey (see also: Nelson et al. 2011; Nomura et al. 2011), with shorter mouthing durations in the second compared to the first trial despite no change in the frequency of fish attacks. Additionally, the overall duration of prey mouthing declined with an increasing number of attacks during subsequent trials. The short experiment duration did not allow inference of long-term retention of the memory of toxic prey. The decrease in prey handling time was not necessarily a result of learning; when sampling the same group of toad tadpoles, the predators may have been exposed to increasing amounts of defensive toxins as tadpoles were repeatedly captured and possibly injured. However, we observed a decrease in mouthing time from the first to the second trial, despite a fresh group of tadpoles being used in the second trial. This indicates that the change in fish behaviour was based on learning and not a simple aversion to increasingly toxic prey. A continuous yet cautious sampling of prey and rejecting the unpalatable individuals has been documented as a way to discriminate between automimics and models in aposematic systems (Guilford 1994; Gamberale-Stille and Guilford 2004; Holen 2013). An obvious benefit for the predator is that this strategy reduces the exposure to prey toxins (Gamberale-Stille and Guilford 2004) and limits the opportunities for cheating by palatable mimics (Skelhorn and Rowe 2006). However, prey is more likely to escape if predators treat it with caution (Sherratt 2002; Yamazaki et al. 2020). The costs for unpalatable prey when being attacked and tasted are still unclear, as well as the fitness value of conspicuous traits if they do not deter predators from sampling the prey (Rowland et al. 2010). In laboratory experiments, vertebrate predators have been observed to taste and reject bufonid tadpoles, apparently unharmed (Peterson and Blaustein 1991; D’Heursel and Haddad 1999; Crossland 2001; Grasso et al. 2010). However, in contrast to animals morphologically adapted to being handled by predators (Sillén-Tullberg 1985; Skelhorn and Rowe 2006; Wang et al. 2018), anuran tadpoles are highly sensitive to handling due to their delicate skin, with even slight injuries possibly leading to mortality during repeated attacks (Duellman and Trueb 1986). In the present study, a few toad tadpoles died after being captured, although they were not consumed. However, the decrease in the duration of prey mouthing suggests that experienced fish treat tadpoles with increasing caution (Nelson et al. 2011; see also: Paradise and Stamp 1991; Hotová Svádová et al. 2013), which could mitigate the injury risk for unpalatable prey (Sillén-Tullberg et al. 1982; Paradise and Stamp 1991). Indeed, toad tadpoles had higher survival rates during the second than the first trial. Decreasing recognition time (Hughes 1979) and increasing caution may explain the persistence of the unpalatable prey when predators choose to sample them despite their conspicuous colouration, i.e., why natural (individual) selection does not act against aposematic prey (see also: Wiklund and Järvi 1982). Occurrence at high densities (Gazzola and Van Buskirk 2015) and gregariousness of unpalatable prey (Waldman and Adler 1979; Svádová et al. 2014) may provide fitness benefits additional to improved learning by predators (Skelhorn et al. 2016), in that costs of sampling by predators are spread over more conspecifics (density-dependent dilution; Speed 2000; Rowland et al. 2010).

## Supplementary Information

Below is the link to the electronic supplementary material.

Supplementary file1 (DOC 467 KB)

## Data Availability

Raw data from the study can be accessed in the electronic supplementary material S2.
