# Supplementary material for: Increasingly cautious sampling, not the black colouration of unpalatable prey, is used by fish in avoidance learning
Source: Anim Cogn. 2023 Jul 28;26(5):1705–11. doi: 10.1007/s10071-023-01815-9 (PMC10442269; doi:10.1007/s10071-023-01815-9)
Supplement: Supplementary file 1 — Supplementary file1 (DOC 467 KB) [file 10071_2023_1815_MOESM1_ESM.doc]

Supplementary Information I

**Materials and methods**

***Model species - transient albinism***

In amphibians, the inability to synthesize dark pigment typically originates from single, recessive mutations (Dubois and Henle 2017; Henle et al. 2017a). In transient albinism, tadpoles developing from white eggs gradually darken during development (Henle et al. 2017b) (Fig. S1).


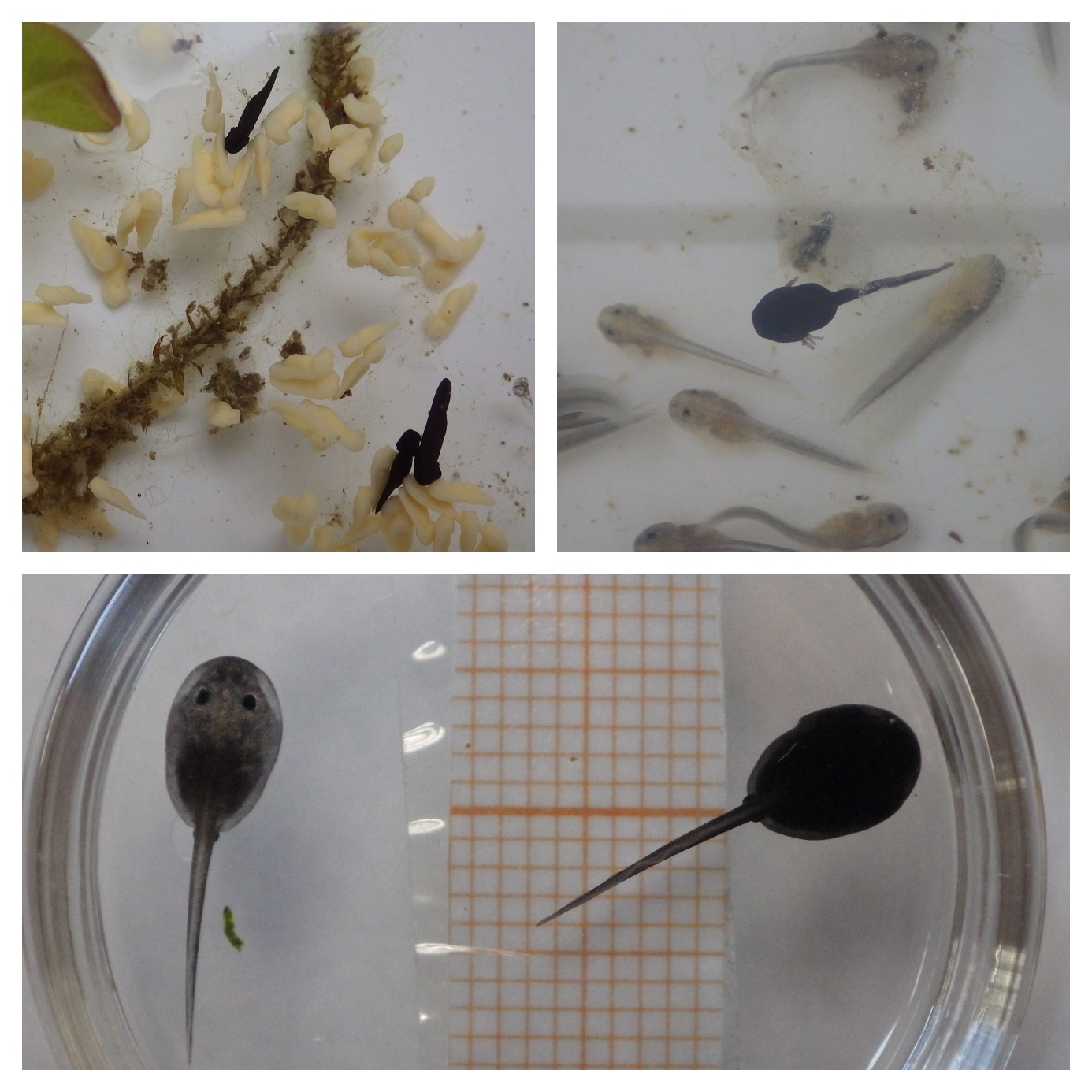


**Fig. S1.** Early development of two common toad *Bufo bufo* phenotypes: transient albino and normal, uniformly black pigmented: larvae (top; Gosner stage 20, 23) and tadpoles used in the present study (bottom; stage 25).

***Analysis of toxin content***

Ten tadpoles from each phenotype were randomly selected, chilled and preserved at -25°C. Tadpoles were homogenized for 1 min with 1 mL of methanol. The samples were centrifuged at 15000 × g, and the supernatant was transferred to new tubes. The obtained solutions were diluted, and five bufadienolide compounds, bufalin, bufotalin, cinobufagin, cinobufotalin and resibufogenin, were identified using commercially acquired reference standards (Merck, Poznań, Poland). These bufadienolides are the most common and abundant compounds in toxic skin secretions of bufonid toads (Zhan et al. 2020).

Liquid chromatography-electrospray ionization tandem mass spectrometric (LC-ESI-MS/MS) analysis was carried out using a Shimadzu UHPLC Nexera XR system equipped with a binary pump coupled with a triple-quadrupole mass spectrometer (LCMS-8045, Shimadzu Corp., Kyoto, Japan). Chromatographic separations were achieved on an ASCENTIS EXPRESS C18 column (100 mm × 2.1 mm, 2.7 µm particle size, Supelco, Inc.). The analytical column was maintained at 35°C and eluted with a mobile phase consisting of (A) water containing 0.1% formic acid and (B) acetonitrile using the following gradient program: 0-8 min (20% B), 8-9 min (60% B), 9-10.5 min (95% B) and 10.5-13 min (20% B) at a flow rate of 0.35 ml/min. The total run time was 13 min with a 2.5 min equilibration time, and the injection volume was 5 µl. Data collection, peak integration, and calculations were performed using the LabSolutions LCMS software (Shimadzu Corp., Kyoto, Japan). The quantity of each compound was estimated from the area under the multiple reaction monitoring (MRM) chromatogram peaks based on the calibration curve of each bufadienolide standard. Compound-dependent parameters used in toxin identification are listed in Table S1.

**Table S1.** Tandem mass spectrometry (MS/MS) transitions and parameters for the detection of bufadienolides in *Bufo bufo* tadpoles

| Compound | Retention time (min) | Multiple reaction monitoring (MRM) transitions (*m/z*) | Collision energy (V) |
| --- | --- | --- | --- |
| Bufalin | 7.83 | 387.4-107.2  387.4-105.1 | 37  53 |
| Bufotalin | 6.22 | 445.1-349.3 | 20 |
| Cinobufagin | 7.84 | 443.1-105.0  443.1-187.2 | 15  14 |
| Cinobufotalin | 6.51 | 459.4-363.3  459.4-381.2 | 21  19 |
| Resibufogenin | 7.85 | 385.4-105.1 | 46 |

References

Dubois A, Henle K (2017) Methodological recommendations for studying the causes of anomalies in natural populations of amphibians. In: Henle K, Dubois A (eds) Studies on anomalies in natural populations of amphibians. Mertensiella, Mannheim, pp 243–260

Henle K, Dubois A, Vershinin V (2017a) Commented glossary, terminology and synonyms of anomalies in natural population of amphibians. In: Henle K, Dubois A (eds) Studies on anomalies in natural populations of amphibians. Mertensiella, Mannheim, pp 9–48

Henle K, Dubois A, Vershinin V (2017b) A review of anomalies in natural populations of amphibians and their potential causes. In: Henle K, Dubois A (eds) Studies on anomalies in natural populations of amphibians. Martensiella, Mannheim, pp 57–164

Zhan X, Wu H, Wu H, et al (2020) Metabolites from *Bufo gargarizans* (Cantor, 1842): a review of traditional uses, pharmacological activity, toxicity and quality control. J Ethnopharmacol 246:112178. doi: 10.1016/j.jep.2019.112178
